# A Multifunctional Electrocatalyst for Formate Production with Concurrent Hydrogen Evolution and Electrochemical Hydrogenation of Glucose to Sorbitol

**DOI:** 10.1021/acsami.6c07887

**Published:** 2026-07-14

**Authors:** Karol V. Mejia-Centeno, Jesus Chacón-Borrero, Qian Xue, Xueqiang Qi, Sara Martí-Sánchez, Jordi Llorca, Doris Cadavid, Malik Dilshad Khan, Jordi Arbiol, Paulina R. Martinez-Alanis, Andreu Cabot

**Affiliations:** † Catalonia Institute for Energy Research−IREC, Jardins de les Dones de Negre 1, 2a pl., Sant Adrià de Besòs 08930 Spain; ‡ Facultat de Química, Universitat de Barcelona, Carrer de Martí i Franquès, Barcelona 08028, Spain; § College of Chemistry and Chemical Engineering, 232838 Chongqing University of Technology, Chongqing 400054, China; ∥ Catalan Institute of Nanoscience and Nanotechnology ICN2, CSIC and BIST, Campus UAB, Bellaterra, Barcelona 08193, Catalonia, Spain; ⊥ Institute of Energy Technologies, Department of Chemical Engineering, and Center for Research in Multiscale Science and Engineering, Universitat Politècnica de Catalunya, EEBE, Eduard Maristany 10-14, Barcelona 08019, Spain; # Departamento de Física, 28021 Universidad Nacional de Colombia, Ciudad Universitaria, Bogotá 111321, Colombia; ¶ ICREA Pg. Lluis Companys, Barcelona 08010, Catalonia, Spain; ∇ Instituto de Química, Universidad Nacional Autónoma de México, Circuito Exterior, Ciudad Universitaria, Ciudad de Mexico 04510, Mexico

**Keywords:** glucose electrooxidation, core−shell catalyst, electrocatalytic hydrogenation, formic acid, biomass valorization, hydrogen

## Abstract

Electrochemical valorization of biomass-derived glucose offers a promising route for the sustainable coproduction of valuable chemicals and green hydrogen, yet hinges on the development of cost-effective, multifunctional catalysts. Here, we report a noble metal-free core–shell catalyst, Cu@CoFe_2_O_4_, that drives both anodic glucose oxidation and cathodic hydrogen evolution or glucose hydrogenation in a single system. In alkaline media (1 M KOH), the catalyst achieves a glucose conversion of 97.5% with a Faradaic efficiency (FE) of 91.2% toward formate at 1.3 V vs RHE, while enabling hydrogen evolution with 100% FE. In a neutral electrolyte (0.1 M Na_2_SO_4_), the same electrode facilitates the selective electrochemical reduction of glucose to sorbitol with an FE of 87.5% and 76% conversion at −0.4 V vs RHE. Experimental and computational analyses suggest that the Cu-rich core/CoFe_2_O_4_-rich shell-like heterostructure enhances charge transfer and provides complementary active sites, contributing to the observed activity toward both glucose oxidation and hydrogenation pathways. This work highlights a versatile, earth-abundant electrocatalytic platform for biomass conversion and sustainable hydrogen generation.

## Graphical abstract

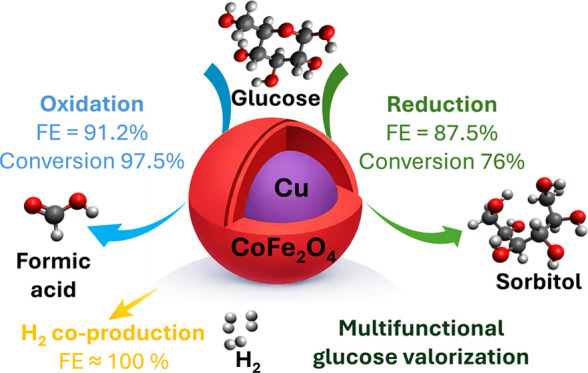

## 1 Introduction

A promising strategy to overcome the energy-intensive nature of water electrolysis involves replacing the anodic oxygen evolution reaction (OER) with the electrooxidation of biomass-derived compounds. These oxidation reactions are thermodynamically more favorable and generate valuable chemicals alongside hydrogen. In this context, glucose, a renewable platform molecule derived from lignocellulosic biomass, has emerged as a model substrate.^1,2^ Its anodic oxidation releases electrons that drive the cathodic hydrogen evolution reaction (HER), enabling the coproduction of green hydrogen and value-added products like formic acid, a versatile chemical and potential hydrogen carrier.

Glucose can also be directly valorized via electrochemical hydrogenation (ECH) in aqueous media. Under neutral conditions, ECH enables the selective reduction of glucose to sorbitol, a commercially important sugar alcohol.^3–5^ This process avoids the need for external hydrogen gas, aligning with the principles of green chemistry, and has garnered significant attention for its efficiency in transforming oxygenated biomass.^6–9^

Integrating these two pathways, anodic glucose oxidation to formate in alkaline media and cathodic glucose hydrogenation to sorbitol in neutral media, within a single bifunctional electrochemical platform represents a particularly attractive goal.^9,10^ Such electrocatalytic strategies can improve energy efficiency and enable the production of value-added chemicals through complementary oxidation and reduction processes under mild conditions (Scheme 1). Realizing this approach, however, requires the development of robust, cost-effective electrocatalysts that maintain high activity, selectivity, and stability across these diverse reaction environments.

**Scheme 1:**
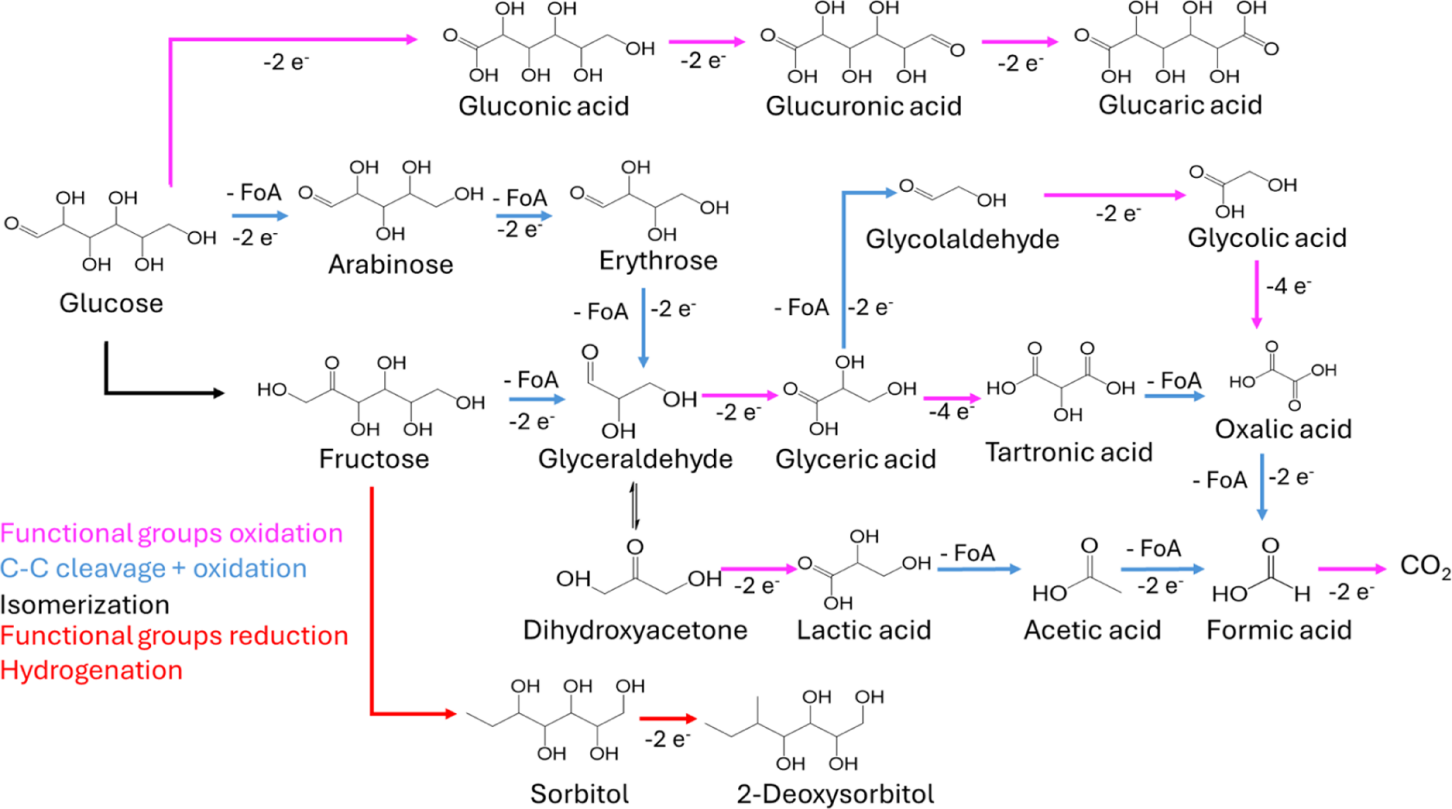
Schematic Overview of Electrocatalytic Processes for GOR and ECH Reactions

While noble metals (e.g., Au, Pt, Pd) show excellent activity for glucose electrooxidation (GOR) and ECH, their high cost and scarcity preclude large-scale application.^11^ In contrast, spinel-type oxides based on earth-abundant cobalt and iron have emerged as promising alternatives due to their redox flexibility and stability.^12^ Although Cu-based catalysts have demonstrated promising activity for glucose electrooxidation and electrochemical hydrogenation, achieving high selectivity and stability across different reaction pathways remains challenging.^13–15^ Similarly, CoFe_2_O_4_ spinels exhibit attractive redox properties and structural robustness, but their relatively low electronic conductivity can limit catalytic performance.^12,16^ Previous studies have predominantly focused on either glucose oxidation or glucose hydrogenation using distinct catalyst systems, whereas reports demonstrating both transformations using a single earth-abundant electrocatalyst remain scarce. Therefore, the development of multifunctional catalysts capable of efficiently promoting both oxidative and reductive glucose conversion pathways remains an important challenge.

In this study, we report a Cu@CoFe_2_O_4_ catalyst with a Cu-rich core/CoFe_2_O_4_-rich shell-like heterostructure supported on carbon cloth (CC) as a multifunctional electrocatalytic platform for glucose valorization. The design combines conductive Cu-rich domains, which facilitate electron transport, with a redox-active CoFe_2_O_4_-rich outer region. This structure enhances charge transfer and provides complementary redox-active sites: Cu­(I)/Cu­(II) couples promote selective glucose oxidation, while Co/Fe centers facilitate hydroxyl activation and intermediate stabilization.^13–15^ This shell-like heterostructural configuration promotes interfacial interactions between the conductive Cu-rich component and the redox-active CoFe_2_O_4_-rich regions, favoring charge transfer and catalytic activity under electrochemical conditions.^16,17^ In alkaline media, the catalyst demonstrates excellent activity and selectivity for GOR to formate with concomitant hydrogen generation. Remarkably, under neutral conditions, the same material enables the selective ECH of glucose to sorbitol, confirming its versatile bifunctionality. These findings highlight the potential of rationally designed, earth-abundant heterostructures as efficient electrocatalysts for sustainable biomass valorization and hydrogen coproduction.

## 2 Experimental Section

### 2.1 Catalyst Synthesis

A series of cobalt-, iron-, and copper-based catalysts were synthesized via a hydrazine-mediated reduction method in ethylene glycol (EG), adapted from previously reported procedures.^18–20^ The synthesis of the optimal composition, with a metal molar ratio of Co/Fe/Cu = 1:1:2, is described as representative. First, stoichiometric amounts of CoCl_2_·6H_2_O (0.3 mmol), FeCl_3_ (0.6 mmol), and CuCl_2_·2H_2_O (0.3 mmol) were dissolved in 20 mL of EG, which acted as both solvent and nanoparticle stabilizer. The solution was heated to 60 °C under an argon atmosphere with continuous stirring. Subsequently, 1 mL of an aqueous 0.5 M hydrazine hydrate solution and 10 mL of a 1.0 M NaOH solution in EG were added dropwise over approximately 30 min. Upon hydrazine addition, the initial brown solution gradually darkened, indicating nanoparticle nucleation. The mixture was maintained at 60 °C for 120 min, then cooled to room temperature. The resulting solid product was collected by centrifugation (7500 rpm for 5 min), washed three times with ethanol, and dried overnight at 60 °C under vacuum. To elucidate the role of each metal component in forming this structure, three control catalysts, FeCuO_*x*_, CoCuO_*x*_, and CoFeO_*x*_, were synthesized. These were prepared by selectively omitting the precursor of one metal (cobalt, iron, or copper, respectively) while keeping the total metal ion concentration constant, following the same hydrazine-mediated procedure. Notably, under these specific conditions, the CoFeO_*x*_ sample did not form stable nanoparticles or a visible precipitate, highlighting the critical role of copper in the nucleation and growth process that yields the defined core–shell morphology.

### 2.2 Computational Method

Spin-polarized density functional theory (DFT) calculations were performed using the Vienna Ab initio Simulation Package with the Perdew–Burke–Ernzerhof exchange–correlation functional.^21^ The core electrons were treated using the projector augmented wave method. A cutoff energy of 450 eV was used for the plane-wave basis set, and a self-consistent-field energy convergence threshold of 1 × 10^–5^ eV/atom was applied. The convergence criterion for structural optimization was a maximum force of 0.02 eV/Å and a maximum displacement of 0.001 Å. The *k*-point meshes were set to 2 × 2 × 1 using the Monkhorst–Pack method, appropriate for the size of the slab model.^22^

Surface models of the Cu@CoFe_2_O_4_ catalyst were constructed to represent its core–shell architecture, with a metallic Cu core and an outer CoFe_2_O_4_ spinel shell. Two representative low-index crystallographic surfaces (111) and (211) were created based on HRTEM observations. The (111) surface was used to simulate glucose oxidation under alkaline conditions (1 M KOH + 0.1 M glucose), while the (211) surface was used to simulate glucose reduction under neutral conditions (0.1 M Na_2_SO_4_ + 0.1 M glucose). A vacuum layer of 15 Å was added along the *z*-direction to eliminate interslab interactions.

The adsorption energy (*E*_a_–*d*_s_) of glucose and other intermediates on the catalyst surfaces was calculated using eq 1

1 $$E_{ads}=E_{composite+adsorbate}-E_{composite}-E_{adsorbate}$$

where *E*_composite+adsorbate_, *E*_composite_ and *E*_adsorbate_ are the total energy of the adsorption, catalyst system, the clean surface, and the isolated glucose molecule, respectively. A more negative value indicates a stronger adsorption on the surface.

### 2.3 Product Quantification

Product quantification was carried out via high-performance liquid chromatography (HPLC). The reaction mixture was acidified to pH ≈ 1 to ensure that the oxidation products of glucose were present in their fully protonated (acid) form. This acidification step is justified by the p*K*_a_1__ values of the main carboxylic acid products, namely formic acid (p*K*_a_1__ = 3.7), oxalic acid (p*K*_a_1__ = 1.27), gluconic acid (p*K*_a_1__ = 3.7), and glucaric acid (p*K*_a_1__ = 3.6). Under these strongly acidic conditions (pH < p*K*_a_1__), the equilibrium is shifted toward the nondissociated acid species, facilitating reliable chromatographic separation and accurate quantification by HPLC.

## 3 Results and Discussion

### 3.1 Catalyst Synthesis and Characterization

The overall synthetic approach and design rationale for the catalyst are illustrated in Scheme 2. In this strategy, hydrazine served as a strong reducing agent, enabling the rapid reduction of Cu^2+^ ions to metallic Cu, which nucleated first to form the conductive core. The presence of NaOH promoted hydrolysis and subsequent condensation of Co^2+^ and Fe^3+^ species, which crystallized as a CoFe_2_O_4_ spinel shell around the preformed copper nuclei. Ethylene glycol (EG) acted simultaneously as a solvent, stabilizer, and coordinating medium, suppressing uncontrolled aggregation and facilitating the controlled growth of the shell.

**Scheme 2:**
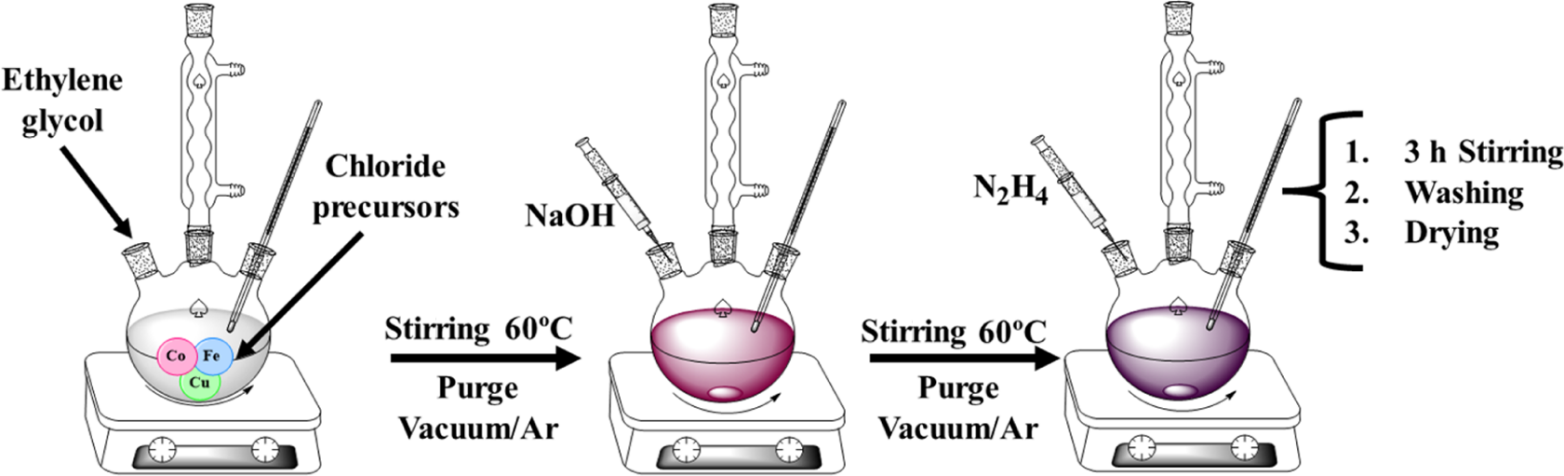
Schematic Diagram for the Preparation of the Catalysts

X-ray diffraction (XRD) patterns of the Cu@CoFe_2_O_4_ catalyst (Figure 1a) are dominated by sharp reflections of metallic Cu (2θ ≈ 43.3° and 50.4°; JCPDS No. 00-004-0836), while only weak, broad features are ascribable to the CoFe_2_O_4_ spinel phase. This Cu-dominated signal reflects the higher crystallinity and diffracting volume of the metallic component. In contrast, the FeCuO_*x*_ and CoCuO_*x*_ control samples display additional reflections corresponding to segregated phases like Fe_2_O_3_ (JCPDS No. 00-040-1139), suggesting that the ternary system favors the formation of a Cu-rich core/CoFe_2_O_4_-rich shell-like heterostructure rather than segregated oxide phases.

**Figure 1:**
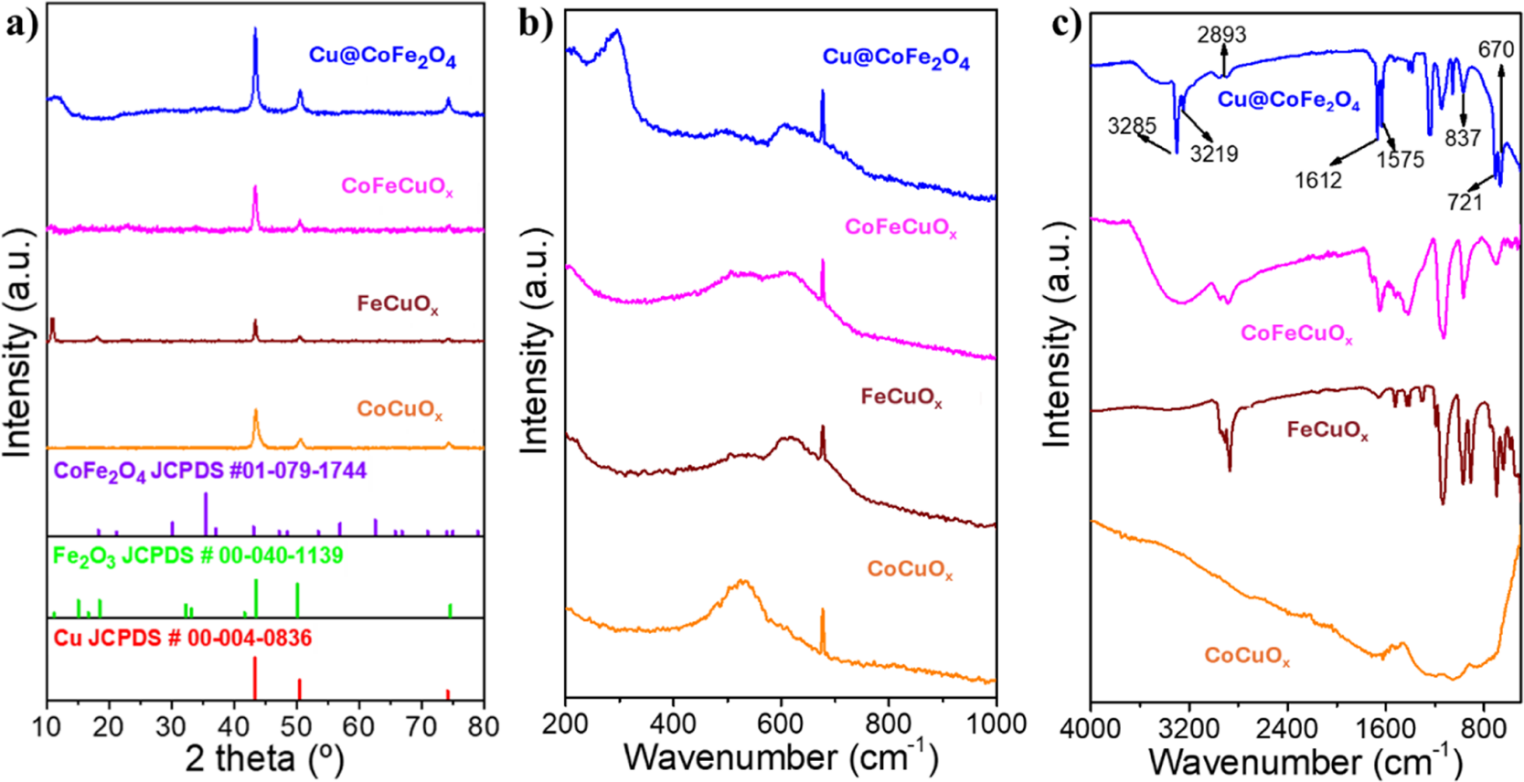
(a) XRD, (b) Raman, and (c) FTIR analysis of Cu@CoFe_2_O_4_ and related materials.

To gain complementary structural insight, Raman spectroscopy was employed (Figure 1b). A strong band around ∼530 cm^–1^ is assigned to Cu–O vibrations at the interface of metallic copper with the spinel shell.^16,23^ Critically, additional bands in the ranges of ∼190–300 cm^–1^ and ∼620–700 cm^–1^ correspond to E_g_ and A_1g_ vibrational modes of the CoFe_2_O_4_ spinel lattice,^24^ which are more clearly resolved by this technique due to its sensitivity to local vibrations. The coexistence of Cu-specific and spinel-related features thus supports the composite structure. Moreover, the absence of significant peaks from Fe_2_O_3_ or CoO phases suggests that ternary incorporation suppresses the phase segregation observed in the binary systems.

Fourier-transform infrared (FTIR) spectra of the Cu@CoFe_2_O_4_ catalyst (Figure 1c) further corroborate the presence of the oxide phase despite the Cu-dominated XRD pattern. Characteristic metal–oxygen stretching vibrations of the CoFe_2_O_4_ spinel lattice are observed at approximately 670, 721, and 837 cm^–1^, in good agreement with previous reports.^25,26^ The bands at 1600, 950, 3285, and 3219 cm^–1^ are assigned to C=O and NH_3_-related stretching modes from residual surface groups generated during the hydrazine/base synthetic process. The weak contribution of residual species indicates a relatively clean metal–oxide interface.^27,28^ Furthermore, no distinct features attributable to CoCuO_*x*_ are observed, which may be related to the low structural order of mixed Co–Cu oxides, typically giving rise to weak and broadly distributed vibrational modes.

Finally, elemental analysis by inductively coupled plasma optical emission spectroscopy (ICP–OES) (Table S1) confirmed the efficient incorporation of Co, Fe, and Cu, with the overall composition closely matching the intended Co/Fe/Cu molar ratio, verifying the successful integration of all three precursors during synthesis.

The morphology and elemental distribution of the as-synthesized catalysts were directly visualized using electron microscopy. Scanning electron microscopy (SEM) images (Figure S1) reveal that all samples consist of agglomerated nanoparticulate domains with rough surface textures, a favorable characteristic for electrocatalysis.^29^ A qualitative comparison indicates that the ternary Cu@CoFe_2_O_4_ catalyst (Figure S1a) exhibits a more uniform particulate morphology, whereas the bimetallic FeCuO_*x*_, CoCuO_*x*_, and CoFeCuO_*x*_ control samples (Figure S1b–d) display more irregular and heterogeneous textures. This observed difference in morphology may reflect the different growth and assembly processes occurring in the ternary and binary systems.

The phase and atomic distribution of the Cu@CoFe_2_O_4_ catalyst was further elucidated using transmission electron microscopy (TEM). High angle annular dark field (HAADF) Scanning TEM (STEM) imaging (Figure 2a) revealed aggregated nanoparticles with uneven contrast, indicative of a heterogeneous composition. Elemental mapping (Figure 2b) and line-scan analysis (Figure S3) provided evidence supporting a shell-like heterostructural arrangement. The Cu signal is preferentially enriched in localized inner regions, whereas Co and Fe are more broadly distributed and contribute significantly to the oxide-rich outer regions, consistent with the formation of a Cu-rich core/CoFe_2_O_4_-rich shell-like heterostructure. It should be noted that the EELS measurements were performed using a Cu TEM grid, which contributes to the overall Cu signal. Nevertheless, the relative spatial distribution of the elements remains discernible and supports preferential Cu enrichment within the particles rather than a simple homogeneous mixed oxide.

**Figure 2:**
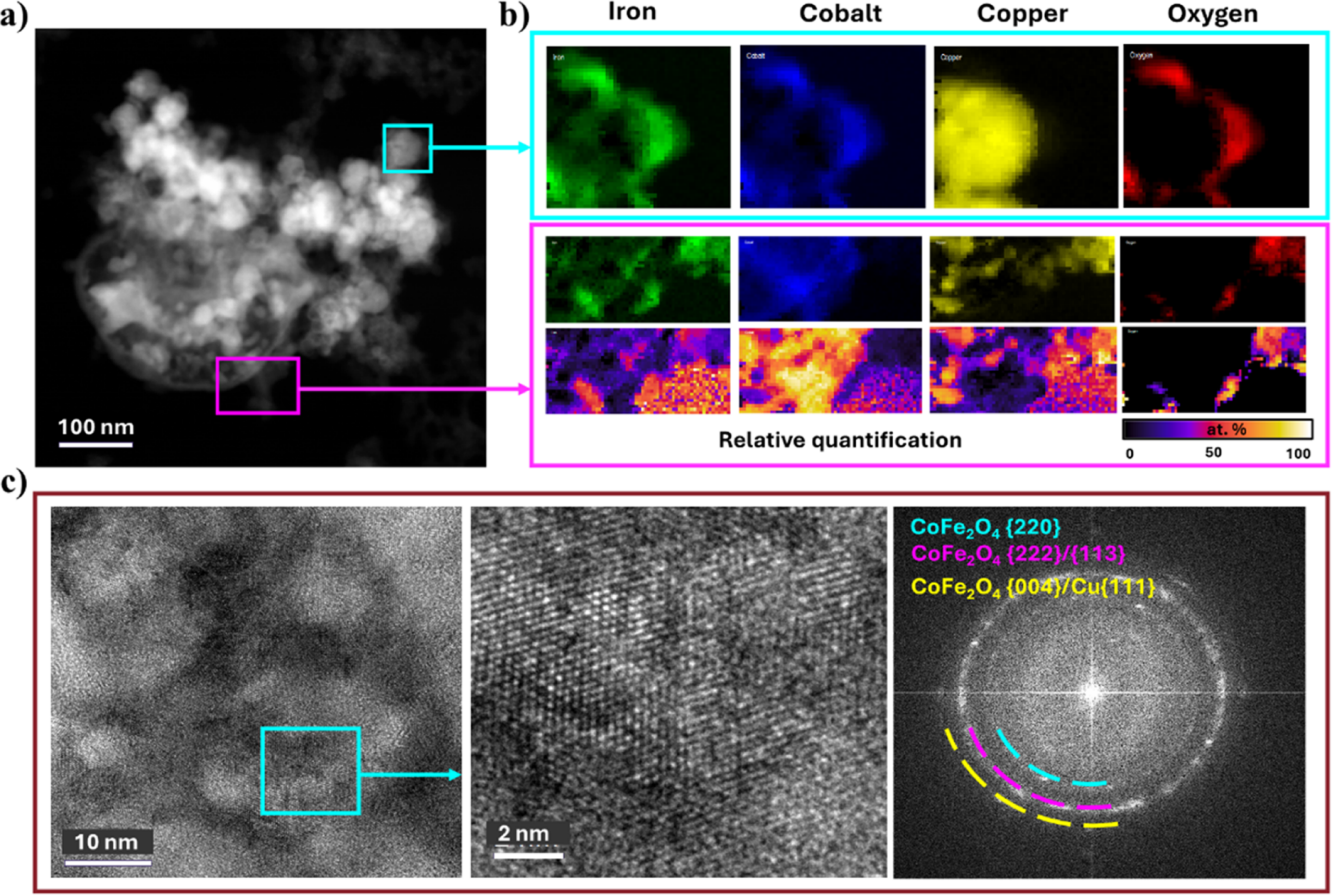
(a) HAADF STEM general view image of Cu@CoFe_2_O_4_. (b) STEM EELS composition maps of Cu@CoFe_2_O_4_ particles showing Co (blue), Fe (green), Cu (yellow), and O (red) distributions. (c) Lattice-resolved HRTEM image and detail of the selected region with its corresponding FFT pattern showing the CoFe_2_O_4_ and Cu^0^ planes.

High-resolution TEM (HRTEM) imaging and the corresponding fast Fourier transform (FFT) pattern (Figure 2c) confirmed the coexistence of crystalline phases. Measured interplanar spacings of 0.296 and 0.253 nm were indexed to the {220} and {222}/{113} planes of the CoFe_2_O_4_ spinel, respectively. An additional spacing of 0.208 nm can be assigned to the {111} plane of metallic Cu and may also overlap with the {004} reflection of CoFe_2_O_4_, supporting the coexistence of Cu and spinel oxide domains.

X-ray photoelectron spectroscopy (XPS) was employed to probe the surface composition and oxidation states. Because XPS is surface-sensitive, this result mainly reflects the oxidized surface environment and does not exclude the presence of metallic Cu-rich domains in the particle interior, as supported by XRD and HRTEM. Under electrochemical operation, Cu species may dynamically evolve among Cu^0^, Cu^+^, and Cu^2+^ states depending on the applied potential and local reaction environment.^30,31^ The Co 2p_3/2_ spectrum (Figure S4b), with a peak at ∼780.4 eV and a satellite at ∼786.0 eV, confirmed the presence of Co^2+^ in octahedral sites of the spinel lattice. Similarly, the Fe 2p_3/2_ signal (Figure S4c), centered at ∼710.6 eV, was assigned to Fe^3+^ in octahedral coordination. The O 1s spectrum (Figure S4d) was fitted with three components. The main peak at ∼531.3 eV is attributed to lattice oxygen (O_lattice_), while the contribution at ∼531–535 eV corresponds to adsorbed oxygen species (O_ads_). Nitrogen adsorption–desorption isotherms were used to investigate the textural properties of the catalysts (Figure S6 and Table S2). The specific surface areas calculated using the Brunauer–Emmett–Teller (BET) method, revealed significant differences between the samples. The CoFeCuO_*x*_ control material exhibited the highest surface area of 10.07 m^2^ g^–1^, whereas the Cu@CoFe_2_O_4_ catalyst showed a lower area of 3.54 m^2^ g^–1^. The average pore diameters, estimated via the Barrett–Joyner–Halenda (BJH) model, were 20.85 nm for CoFeCuO_*x*_ and 27.35 nm for Cu@CoFe_2_O_4_. This variation in textural properties is consistent with their differing structural evolution: the core–shell architecture of Cu@CoFe_2_O_4_ likely leads to denser particle packing and fewer exposed mesopores compared to the more porous, presumably less-ordered aggregate structure of the ternary oxide control sample.

### 3.2 Electrocatalytic Performance

The electrocatalytic activity of the synthesized catalysts toward the GOR was investigated in a 1.0 M KOH electrolyte containing 0.1 M glucose using a standard three-electrode configuration with a platinum gauze counter electrode, an Hg/HgO reference electrode, and 10 mg of the supported catalyst as the working electrode. Figure 3a compares the linear sweep voltammetry (LSV) profiles of FeCuO_*x*_, CoCuO_*x*_, CoFeCuO_*x*_, and Cu@CoFe_2_O_4_ on glassy carbon electrodes. The Cu@CoFe_2_O_4_ catalyst exhibited the highest activity, achieving a current density exceeding 300 mA cm^–2^ at 2.0 V vs RHE, outperforming the bimetallic analogues. This superior performance is attributed to cooperative effects between the Co, Fe, and Cu active sites.^32^ For quantitative benchmarking, the potential required to reach 10 mA cm^–2^ was 1.38 V vs RHE for Cu@CoFe_2_O_4_, confirming its superior electrocatalytic performance relative to the control samples.

**Figure 3:**
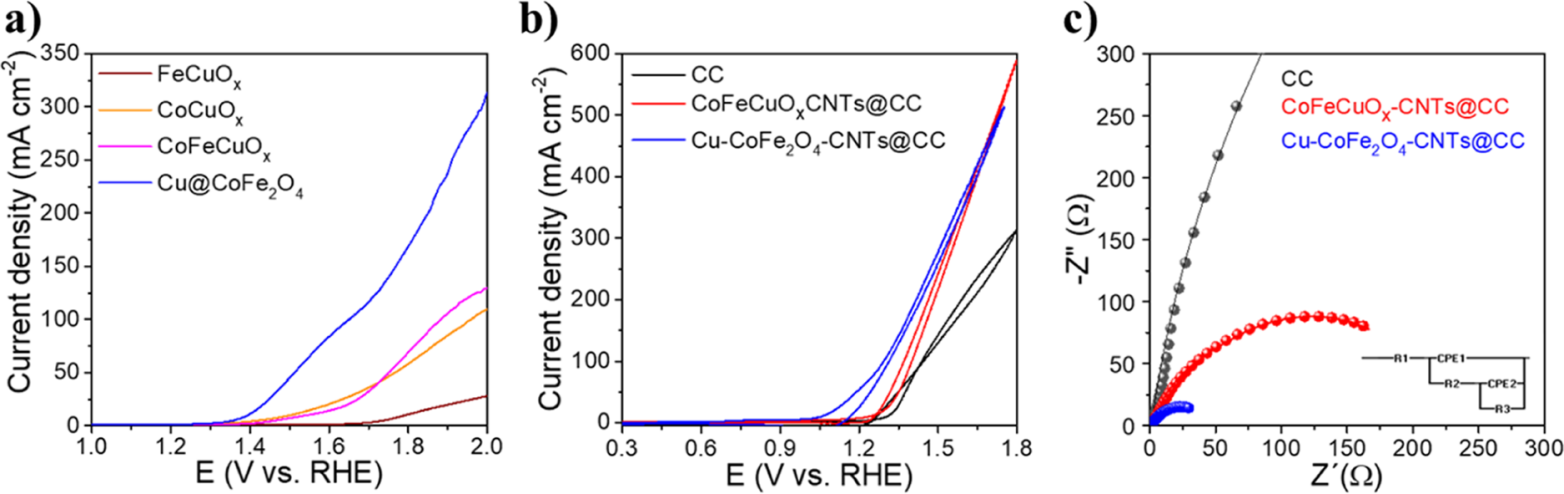
(a) LSV curves of FeCuO_*x*_, CoCuO_*x*_, CoFeCuO_*x*_, and Cu@CoFe_2_O_4_ on GC. (b) CV curves of CC, CoFeCuO_*x*_–CNTs@CC, and Cu@CoFe_2_O_4_–CNTs@CC. (c) Nyquist plots at onset potential in 1.0 M KOH + 0.1 M glucose.

To better emulate practical conditions and enhance electronic conductivity, the catalysts were deposited onto CC and modified with carbon nanotubes (CNTs). SEM images (Figure S2a–c) show that while bare CC fibers are smooth, the Cu@CoFe_2_O_4_ catalyst uniformly coats the surface. The incorporation of CNTs creates a more textured, porous network, facilitating improved interfacial contact and mass transfer. As shown in Figure 3b, this optimized electrode architecture led to a further enhancement in activity. The Cu@CoFe_2_O_4_–CNTs@CC electrode delivered the highest catalytic response, surpassing the performance of both bare CC and the CoFeCuO_*x*_–CNTs@CC control, underscoring the importance of both intrinsic catalyst composition and electrode engineering.^33^

The charge-transfer kinetics at the electrode–electrolyte interface were evaluated using electrochemical impedance spectroscopy (EIS) at the respective onset potentials. The Nyquist plots in Figure 3c were fitted with a modified equivalent circuit model comprising three resistive components (R_1_, R_2_, R_3_) and two constant phase elements (CPE_1_, CPE_2_). The Cu@CoFe_2_O_4_–CNTs@CC electrode exhibited significantly lower charge-transfer resistances (R_2_ = 9 Ω; R_3_ = 31 Ω) compared to the CoFeCuO_*x*_–CNTs@CC control (R_2_ = 50 Ω; R_3_ = 182 Ω) and bare CC (R_2_ = 310 Ω; R_3_ = 2450 Ω). This substantial reduction in interfacial resistance highlights the superior charge-transfer kinetics and enhanced catalytic efficiency of the optimized electrode, which is consistent with reports linking lower Rct values to higher conductivity and electrocatalytic activity.^34^

The electrochemical surface area (ECSA) was estimated from cyclic voltammograms (CV) recorded in a nonfaradaic potential window (0.50–0.60 V vs RHE) at scan rates from 20 to 100 mV s^–1^ (Figure S6). The double-layer capacitance (*C*_dl_) was calculated from the linear slope of the average current plotted against the scan rate (ν), according to eq 2 ^35^

2 $$i_{avg}=\frac{\left|i_{a}\right|+\left|i_{c}\right|}{2}=C_{dl}v$$

From *C*_dl_, the ECSA was then derived using eq 3

3 $$ECSA=C_{dl}/C_{s}$$

where *C*_s_ is the specific capacitance. A standard value of *C*_s_ = 0.04 mF cm^–2^ was used for metal oxides in alkaline media.^9,36^ The calculated ECSA was 0.80 cm^2^ for Cu@CoFe_2_O_4_–CNTs@CC and 0.75 cm^2^ for the CoFeCuO_*x*_–CNTs@CC control.

### 3.3 Coupled Redox Reactions

Having established the superior catalytic activity and favorable charge transfer properties of the Cu@CoFe_2_O_4_–CNTs@CC electrode, we next evaluated its stability and product selectivity during glucose oxidation. Figure 4a illustrates the dual-compartment H-type cell employed for the experiments, configured in a standard three-electrode setup. The two chambers were separated by a Nafion 117 proton-exchange membrane to prevent product crossover. The Cu@CoFe_2_O_4_–CNTs@CC catalyst served as the working electrode in the anodic chamber containing 1.0 M KOH and 0.1 M glucose, where glucose oxidation occurred, while hydrogen evolution simultaneously proceeded at the platinum cathode in the other compartment. Chronoamperometry (CA) experiments were carried out for 8 h at ambient temperature, applying anodic potential between 1.2 and 1.5 V vs RHE (Figure 4b). Below 1.2 V vs RHE, only minor anodic currents were detected, consistent with sluggish glucose conversion, whereas at potentials beyond 1.5 V vs RHE, the OER became increasingly dominant, significantly diminishing the Faradaic efficiency (FE) for valuable products.

**Figure 4:**
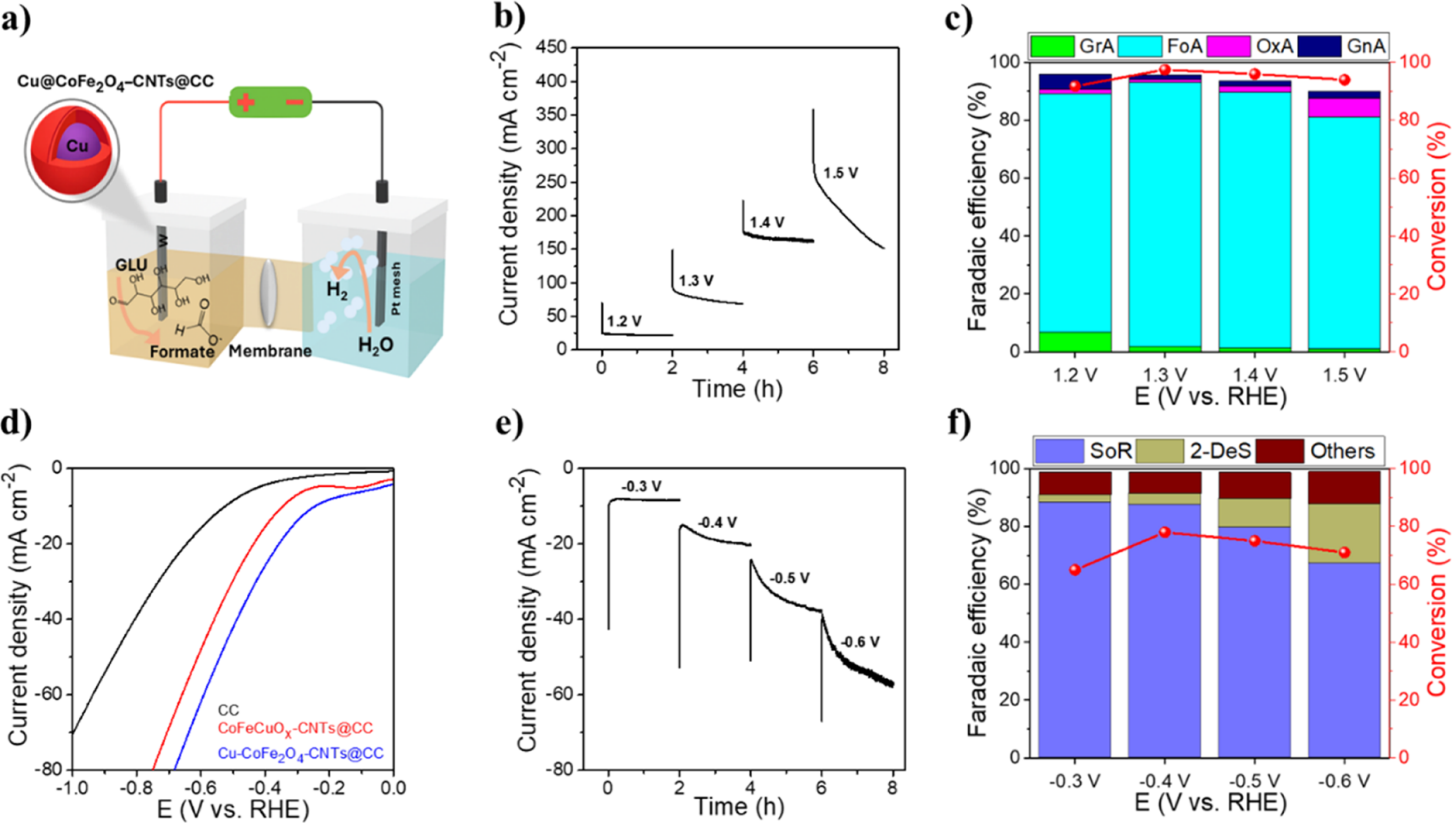
(a) Schematic representation of the overall system coupling glucose oxidation to FoA (anodic side) and hydrogen evolution (cathodic side), enabled by the Cu@CoFe_2_O_4_ catalyst. (b) CA profiles for the GOR at 1.2–1.5 V vs RHE over 8 h, showing increasing current densities with applied potential. (c) Measured FE and glucose conversion. (d) LSV curves comparing CC, CoFeCuO_*x*_–CNTs@CC, and Cu@CoFe_2_O_4_–CNTs@CC electrodes in 0.1 M Na_2_SO_4_ and 0.1 M glucose. (e) CA profiles for the ECH of glucose at −0.3 to −0.6 V vs RHE over 8 h. (f) FE and glucose conversion toward reduction products.

The GOR mechanism involves a series of oxidative steps combining electron transfer and chemical transformations. At lower overpotentials, glucose is oxidized at the aldehyde group (C1) to form gluconic acid, which exists predominantly as gluconate under alkaline conditions and may further convert to gluconolactone depending on the electrode environment. Concurrent oxidation of the hydroxymethyl group at C6 yields glucuronic acid, which can be further transformed into glucaric acid.^37^ At moderately higher potentials, C–C bond cleavage, particularly between the C2–C3 or C3–C4 positions, leads to the formation of short-chain carboxylates such as formate and oxalate, depending on the electrolyte pH.

These products typically arise from deep oxidation steps and reflect a more aggressive oxidative regime. Notably, formate is favored at intermediate potential where OER activity remains suppressed, highlighting the importance of precise potential control.^38–40^

To better understand the preferential route toward formate on our catalyst, the following simplified mechanism is proposed

4 $$M-OH+{OH}^{-}\to M-OOH+H_{2}O+e^{-}$$

5 glucose+M−OOH→glucaricacid+M−OH

6 $$glucaricacid+M-OOH+{OH}^{-}\to {HCOO}^{-}+{CO}_{2}+M-OH+e^{-}$$

Here, the formation of the M–OOH intermediate constitutes the rate-limiting step. The CoFe_2_O_4_ spinel shell offers abundant redox-active sites (Co^3+^/Co^2+^ and Fe^3+^/Fe^2+^), while the Cu core improves electrical conductivity and charge transfer. This synergistic effect helps account for the observed high current density, long-term stability, and selective formate formation. Compared to monometallic systems, the Cu@CoFe_2_O_4_ catalyst demonstrates greater formate selectivity and Faradaic efficiency due to these synergistic effects between the conductive core and redox-active shell. These results highlight the importance of the specific structural organization and interfacial interactions between the Cu-rich core and the CoFe_2_O_4_-rich shell, beyond the contribution of elemental composition alone. The Cu-rich component may further promote glucose adsorption and electron delocalization, thereby facilitating C–C bond cleavage while helping to suppress the competing OER within the optimized potential window.

Notably, at 1.3 V vs RHE, after the acidification step, formic acid (FoA) was measured with a FE of 91.2%, while the contributions of glucaric acid (GrA), oxalic acid (OxA), and gluconic acid (GnA) remained below 5% each (Figures 4c and S7). The glucose conversion rate at this potential reached 97.5%, indicating that the Cu@CoFe_2_O_4_–CNTs@CC catalyst promotes highly selective oxidative cleavage of glucose, consistent with literature reports on noble-metal-free systems (Table S3). In contrast, operation at 1.5 V vs RHE led to a rise in the measured OxA (FE = 6.5%), indicating the onset of overoxidation, either via secondary formate oxidation or parallel decomposition pathways. At this relatively high potential, the evolution of CO_2_ was validated by postelectrolysis acidification of the electrolyte with 2 M H_2_SO_4_, which induced noticeable effervescence due to decomposition of the formed carbonate. These findings confirm that the potential window between 1.3 and 1.4 V vs RHE represents the most favorable operational range for selective GOR to formate, minimizing undesired side reactions while maintaining high electrochemical conversion and efficiency.

Hydrogen production was simultaneously monitored at the cathodic compartment during anodic operation. In the applied potential range of 1.3–1.6 V vs RHE, gas chromatography analysis revealed a FE close to 100% for hydrogen evolution, with no detectable side reactions. These results confirm the effective electron utilization at the cathode and validate the dual performance of the system, which can generate both high-value FoA and high-purity hydrogen under mild electrochemical conditions.

In addition to anodic glucose oxidation, the ECH of glucose was investigated using the same Cu@CoFe_2_O_4_–CNTs@CC electrode in a neutral aqueous medium composed of 0.1 M Na_2_SO_4_ and 0.1 M glucose, aiming to promote the selective production of polyhydroxylated compounds, primarily sorbitol (SoR). A conventional three-electrode H-cell setup was employed for these experiments. Prior to the hydrogenation experiments, LSV analyses compared the activity of the Cu@CoFe_2_O_4_–CNTs@CC electrode with bare CC and CoFeCuO_*x*_–CNTs@CC electrodes (Figure 4d), confirming its superior cathodic response. Subsequently, CA measurements were recorded over an 8 h period at constant cathodic potentials ranging from −0.3 to −0.6 V vs RHE using the Cu@CoFe_2_O_4_–CNTs@CC electrode (Figure 4e). This potential window was carefully selected to drive the hydrogenation reaction while minimizing the occurrence of the parasitic HER.^4^

The ECH mechanism begins with an initial electron transfer to the carbonyl carbon of glucose at the C1 position, reducing the aldehyde functional group (−CHO) to a primary alcohol (−CH_2_OH), thus forming SoR via a two-electron, proton-coupled process.^4^ This route is thermodynamically accessible at moderate overpotentials and represents the most direct hydrogenation pathway: (4) glucose +2H^+^ + 2e^–^ → sorbitol. Additionally, glucose can undergo keto–enol tautomerization to yield fructose or mannose intermediates, which are themselves susceptible to reduction through comparable mechanisms, resulting in a diverse mixture of reduced sugars.^41^

At relatively low cathodic potentials (−0.3 V and −0.4 V vs RHE), SoR emerged as the predominant product, accounting for over 85% of the total compounds detected according to HPLC analysis (Figure 4f). However, as the applied bias became more negative (−0.5 V to −0.6 V vs RHE), the formation of 2-deoxysorbitol (2-DeS) increased markedly. This byproduct likely originates from deeper hydrogenation steps involving C–OH group elimination through dehydration-reduction cascades or direct scission of hydroxylated moieties. Minor fractions of other polyols, such as glycerol, mannitol, or lower-molecular-weight intermediates were also detected and grouped as “others”.^4,5^ Mechanistically, the reduction process is governed by mass transport and charge transfer kinetics at the electrode–electrolyte interface. At increasingly negative potentials, HER becomes more competitive, especially on Cu-rich surfaces with high inherent activity toward H^+^ reduction.^42,43^

Despite this, the Cu@CoFe_2_O_4_–CNTs@CC catalyst exhibited optimal selectivity and activity at −0.4 V vs RHE, achieving a FE of 87.5% for SoR and a glucose conversion rate of 76%, surpassing the performance of conventional copper-based systems. This enhanced electrocatalytic behavior is attributed to the synergistic interaction between the conductive Cu-rich domains and the spinel CoFe_2_O_4_-rich regions, which facilitates electron transport and provides suitable surface sites for glucose hydrogenation under neutral pH conditions.^44–46^

### 3.4 DFT Calculations

To study the bifunctional catalytic performance of the Cu@CoFe_2_O_4_ core–shell catalyst, DFT calculations were performed on the two most representative crystallographic planes: the (111) facet, which represents a close-packed, thermodynamically stable surface, and the (211) facet, which represents a more open, high-index surface rich in low-coordination sites. The (111) surface is typically associated with strong adsorption and oxidative activity, whereas the stepped (211) surface favors electron transfer and reduction pathways due to its higher density of undercoordinated atoms.^47,48^ The top and side views of glucose and its oxidation/reduction intermediates adsorbed on these surfaces are shown in Figure 5.

**Figure 5:**
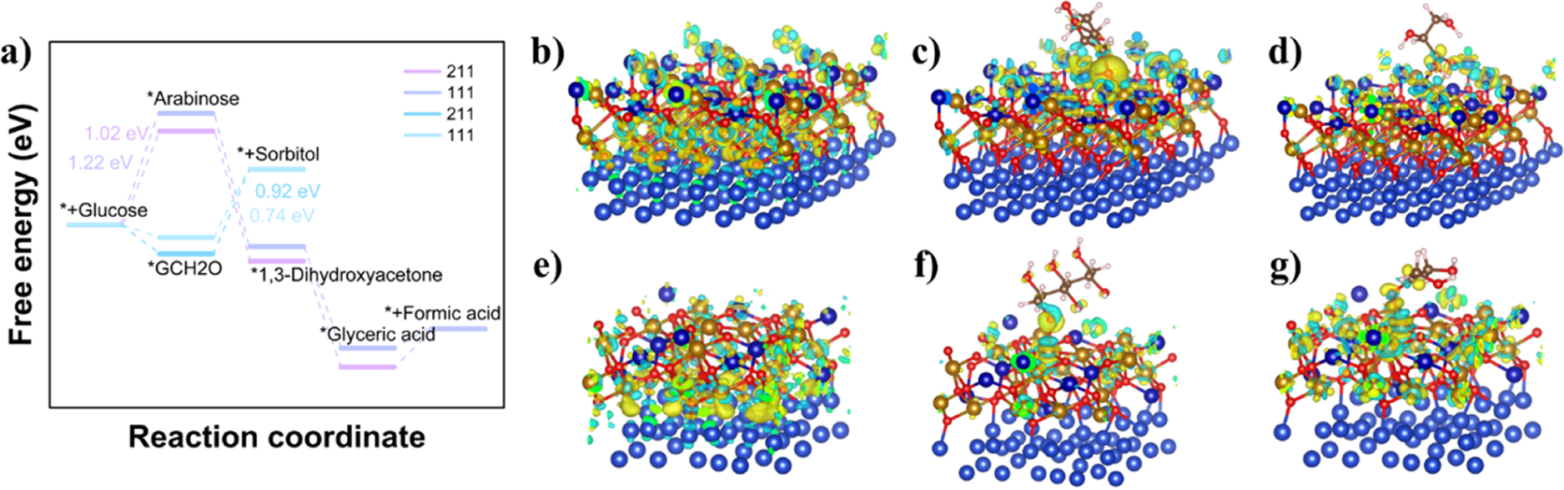
Top and side views of optimized structures of glucose and reaction intermediates adsorbed on Cu@CoFe_2_O_4_ surfaces. The (111) facet under alkaline conditions is shown for: (a) glucose, (b) arabinose, and (c) glyceric acid. The (211) facet under neutral conditions is shown for: (d) glucose, (e) reduced intermediate, and (f) sorbitol. H, C, O, Co, Fe, and Cu atoms are shown in white, brown, red, gold, dark blue, and blue, respectively.

On the (111) surface, which simulates oxidative conditions in alkaline media (Figure 5a–c), glucose exhibits strong adsorption via multiple hydroxyl groups coordinated to surface Fe and Co atoms. The adsorption energy was calculated to be −1.22 eV, indicating a strong interaction. Such strong binding stabilizes the adsorbed molecule and facilitates successive dehydrogenation steps required for oxidation. As oxidation proceeds, glucose is first converted to arabinose and then to glyceric acid, which are thermodynamically favorable products under basic conditions. Spin-polarized PDOS analysis (Figure S8a) shows that the 3d orbitals of Co and Fe atoms shift closer to the Fermi level, particularly for the spin-down states. This increased electronic interaction enables greater charge transfer from the glucose molecule to the surface, promoting oxidative dehydrogenation.

Conversely, on the (211) surface under neutral conditions (Figure 5d–g), glucose exhibits a weaker interaction with the surface, with an adsorption energy of only −0.74 eV. This moderate adsorption prevents overstabilization of intermediates and favors their further reduction and desorption.^49^ In this environment, reduction is favored, and a reduced intermediate with an added hydroxyl group is formed, eventually leading to the formation of sorbitol (Figure 5f,g). The PDOS of this facet (Figure S8b) reveals less overlap between the metal d orbitals and adsorbate states, together with a modest shift of the Cu d-band center toward the Fermi level. These features indicate moderate electron donation from the surface to the adsorbed species, consistent with a reductive pathway.

Additionally, comparing of the spin-polarized d-band centers for Co, Fe, and Cu shows that the (111) facet presents greater spin asymmetry and closer d-band proximity to the Fermi level, suggesting higher oxidative activity. In contrast, the (211) surface, with narrower spin-split d bands and enhanced Cu participation, facilitates electronic conduction and controlled electron donation, supporting reduction reactions. These theoretical results correlate well with the experimental behavior: under alkaline conditions the catalyst selectively oxidizes glucose, whereas under neutral conditions it promotes glucose hydrogenation. Thus, the Cu@CoFe_2_O_4_ catalyst exhibits bifunctional catalytic behavior, which may be influenced by differences in surface structure, with the (111) and (211) surfaces suggested to favor glucose oxidation and reduction pathways, respectively.

## 4 Conclusion

In this work, we report a multifunctional electrocatalytic platform based on a core–shell Cu@CoFe_2_O_4_–CNTs@CC electrode for the electrochemical valorization of glucose. The catalyst enables two distinct, valuable pathways under mild conditions. In alkaline media, it drives the selective anodic oxidation of glucose to formate with a FE of 91.2% and a conversion of 97.5% at 1.3 V vs RHE. Simultaneously, this process facilitates the cathodic HER with nearly 100% FE, enabling the cogeneration of green hydrogen. Under neutral conditions, the same electrode catalyzes the ECH of glucose to SoR with an FE of 87.5% and 76% conversion at −0.4 V vs RHE. The operational potential windows for both reactions were carefully optimized to maximize product selectivity while suppressing competing side reactions such as oxygen evolution and excessive hydrogen evolution. The improved bifunctional performance is attributed to the synergistic design of the Cu@CoFe_2_O_4_ core–shell architecture. The conductive Cu-rich component facilitates charge transfer kinetics, as evidenced by electrochemical impedance spectroscopy. The CoFe_2_O_4_-rich regions provide redox-active sites (Co^2+^/Co^3+^ and Fe^2+^/Fe^3+^) that may contribute to glucose adsorption and stabilization of key reaction intermediate. Comprehensive characterization by XRD, Raman, XPS, and TEM supports the formation of this shell-like heterostructure, while DFT calculations provide a mechanistic rationale by suggesting facet-dependent selectivity: the (111) surface favors oxidative pathways, whereas the (211) surface facilitates reductive transformations. This electronic and structural synergy enhances active site availability, promotes selective C–C bond cleavage for FoA formation, and modulates adsorption for selective hydrogenation to SoR. This work validates a practical strategy for the sustainable coproduction of high-value chemicals (FoA and SoR) and green hydrogen from a single, abundant biomass feedstock using a single, noble-metal-free electrocatalyst. The findings highlight the potential of rationally designed earth-abundant heterostructures to serve as efficient, multifunctional platforms, contributing to the development of sustainable electrochemical biomass conversion technologies within a circular chemical economy.

## Supplementary Material



## Notes

Experimental details; additional SEM, TEM, HRTEM, STEM-EDS, and EELS characterization; XRD and XPS analyses; electrochemical measurements and control experiments; product quantification and calibration curves; DFT calculation details; supplementary figures and tables (PDF)
