# Supplementary material for: A Multifunctional Electrocatalyst for Formate Production with Concurrent Hydrogen Evolution and Electrochemical Hydrogenation of Glucose to Sorbitol
Source: ACS Appl Mater Interfaces. 2026 Jul 14;18(29):39802–11. doi: 10.1021/acsami.6c07887 (PMC13425547; doi:10.1021/acsami.6c07887)
Supplement: Supplementary file 1 [file am6c07887_si_001.pdf]

SUPPORTING INFORMATION

A Multifunctional Electrocatalyst for Formate Production with Concurrent Hydrogen Evolution and Electrochemical Hydrogenation of Glucose to Sorbitol

Karol V. Mejia-Centeno[a,b], Jesus Chacón-Borrero[a], Qian Xue[a], Xueqiang Qi[c], Sara Martí-Sánchez[d], Jordi Llorca[e], Doris Cadavid[f], Malik Dilshad Khan[a*], Jordi Arbiol[d,g], Paulina R. Martinez-Alanis[h*], and Andreu Cabot[a,g*]

a. Catalonia Institute for Energy Research−IREC, Jardins de les Dones de Negre 1, 2ª pl., Sant Adrià de Besòs, Spain.

b. Facultat de Química, Universitat de Barcelona, Carrer de Martí i Franquès, Barcelona 08028, Spain.

c. College of Chemistry and Chemical Engineering, Chongqing University of Technology, Chongqing 400054, China.

d. Catalan Institute of Nanoscience and Nanotechnology ICN2, CSIC and BIST, Campus UAB, Bellaterra, 08193 Barcelona, Catalonia, Spain.

e. Institute of Energy Technologies, Department of Chemical Engineering, and Center for Research in Multiscale Science and Engineering, Universitat Politècnica de Catalunya, EEBE, Eduard Maristany 10-14, 08019 Barcelona, Spain.

f. Departamento de Física, Universidad Nacional de Colombia, Ciudad Universitaria, 111321 Bogotá, Colombia

g. ICREA Pg. Lluis Companys, 08010 Barcelona, Catalonia, Spain.

h. Instituto de Química, Universidad Nacional Autónoma de México, Circuito Exterior, Ciudad Universitaria, 04510 Ciudad de México, México

* mdkhan@irec.cat; paulina.martinez@iquimica.unam.mx; acabot@irec.cat

1. Chemical and reagents

All chemicals used in this experiment were of analytical grade and were used as received without further purification. Iron(III) chloride ($FeCl_3$), cobalt(III) chloride hexahydrate ($CoCl_2 \cdot 6H_2O$), copper(II) chloride dihydrate ($CuCl_2 \cdot 2H_2O$), hydrazine hydrate ($N_2H_4 \cdot H_2O$), and sodium hydroxide (NaOH), D-(+)-glucose (99.5 %), glycolic acid (99 %), D-saccharic acid potassium salt (GrA 98 %), D-(-)-fructose (99 %), oxalic acid (OxA 98 %), D-Sorbitol (SoR, 98%), formic acid (FoA, 97 %) and Nafion (5 wt% in a mixture of low aliphatic alcohols and water) were obtained from Sigma-Aldrich. Lactic acid (LA) solution (90 %) was obtained from VWR, ethylene glycol (EG), sulfato de sodio (Na_2SO_4, 99%), hidróxido de potasio (KOH, 85%) and D-gluconic acid aqueous solution (GnA, 50 %) from Thermo Fisher. Carbon cloth (CC) and carbon nanotubes (CNTs) ware purchased from Carnd-China. Ethanol (96%, analytical grade). MilliQ water was obtained from a Purelab Flex from Elga.

2. Characterization

Powder X-ray diffraction (XRD) patterns were collected from the samples supported on a Si substrate on a Bruker-AXS D8 Advanced X-ray diffractometer with Ni-filtered (2 μm thickness) Cu K radiation (λ = 1.5406 Å) operating at 40 kV and 40 mA. The 2θ range was between 10-80°. Scanning electron microscopy (SEM) analysis was conducted with a Zeiss Auriga microscope equipped with an energy-dispersive X-ray spectroscopy (EDS) detector and backscattering electron (BSE) contrast mode, operating at 20 kV. The element content of the samples was analyzed by Inductively Coupled Plasma-optical emission spectroscopy (ICP-OES, Perkin Elmer Optima 3200RL). High-resolution transmission electron microscopy (HRTEM) images and scanning transmission electron microscopy (STEM) studies were conducted on an FEI Tecnai F20 field emission gun microscope operated at 200 kV with a point-to-point resolution of 0.19 nm, which was equipped with high angle annular darkfield (HAADF) and a Gatan Quantum electron energy loss spectroscopy (EELS) detector. Tip-Enhanced Raman Spectroscopy (TERS) measurements were conducted using an Xplora Nano instrument from HORIBA. A 632 nm laser and Au-coated OMNI TERS tips were utilized for the analysis. Maps were generated with a resolution of 10 nm per pixel, resulting in 350 × 350 nm maps, and plotted using a 1.8-pixel weight average. Acquisition times of 10 seconds per spectrum and a 1200 g mm^{-1} grating were employed during the measurements. Surface areas were calculated using the Brunauer-Emmett-Teller (BET) method, which involves equally spaced points across the P/Po range. Pore size distribution was determined from the desorption branches of the isotherms using the Barrett-Joyner-Halenda (BJH) model. X-ray photoelectron spectroscopy (XPS) was analyzed on a SPECS system equipped with an Al anode XR50 source operating at 150 W and a Phoibos 150 MCD-9 detector. The Multipak data reduction software (Physical Electronic-PHI, Inc., EE. UU.) was employed for the data process (C_{1s} peak corrected at a binding energy of 284.8 eV). Fourier transform infrared (FT-IR) spectra were recorded on an Alpha Bruker spectrometer.

3. Electrochemical tests

3.1 Electrode preparation

A catalyst ink was prepared by ultrasonically dispersing 10 mg of the electrocatalyst in a mixture of 0.8 mL isopropanol, 0.2 mL deionized water, and 50 μL of Nafion solution (5 wt%). The resulting suspension was sonicated for 30 minutes to ensure a homogeneous dispersion. Electrochemical measurements were carried out at room temperature in an H-type

electrochemical cell using a CHI-660E electrochemical workstation. A three-electrode setup was employed, consisting of a Pt gauze as the counter electrode, a Hg/HgO (saturated KOH) electrode as the reference, and a CC, 1 cm^2 as the working electrode. The working electrode was prepared by coating the CC with 0.5 mL of catalyst ink, applied using an aerograph (airbrush) to ensure uniform distribution. The final loading of catalyst on the electrode surface was approximately 5 mg. The resulting electrodes are referred to as Cu-$CoFe_2O_4$–CNTs@CC.

3.2 Electrochemical measurements

Electrocatalytic performance was evaluated using an H-type cell setup at room temperature, connected to a CHI-660E electrochemical workstation. The three-electrode configuration included a platinum (Pt) gauze as the counter electrode, a Hg/HgO reference electrode, and a 1 cm^2 of CC loaded with 10 mg of catalyst as the working electrode. A Nafion 117 proton exchange membrane was used to separate the anodic and cathodic chambers. The cathodic compartment contained 50 mL of 1 M KOH as the electrolyte, while the anodic side was filled with 50 mL of a 10 mM glucose solution in 1 M KOH. Chronoamperometric (CA) measurements were conducted over 2 hours at applied potentials ranging from 1.2 to 1.5 V vs. RHE in the presence of glucose. For ECH studies, the same three-electrode configuration was used, with a neutral aqueous electrolyte composed of 0.1 M Na_2SO_4, and 0.1 M glucose. CA measurements were performed at applied potentials ranging from −0.3 to −0.6 V vs. RHE.

3.3 GOR product analysis

The liquid-phase products generated from the electrooxidation of glucose were analyzed using high-performance liquid chromatography (HPLC), equipped with a Rezex™ ROA-Organic Acid H^+ (8%) column (300 × 7.8 mm), a diode array detector set at 210 nm, and a refractive index detector. The mobile phase used was 0.005 M H_2SO_4, delivered at a flow rate of 0.2 mL min^{-1}, and the column was maintained at 40 ºC during the measurements. Prior to injection, 1 mL of sample was collected and diluted with 0.5 mL of 2 M H_2SO_4. Quantification of the oxidation products was achieved through calibration curves constructed using standard solutions. The Faradaic efficiency (FE) for each product was computed according to Equation (1):

$$FE\ (\%) = \frac{n \cdot F \cdot mol_{product}}{Q_{total}} \cdot 100 \quad (1)$$

where F is the Faradaic constant (96,485 C mol^{-1}), n is the number of transferred electrons, $mol_{product}$ is the mol of each product obtained, and Q_{total} is the total charge passed throughout the experiment.

The conversion of glucose ($\eta_{glucose}$) and yields of its oxidation products (Y_P) were calculated using the following equations (2 and 3):

$$\eta_{glucose} = \frac{1-C_{glucose}}{C_{0-glucose}} \cdot 100\% \quad (2)$$

$$Y_P = \frac{C_p}{C_{0-glucose}} \cdot 100\% \quad (3)$$

where $C_{0\text{-}glucose}$ and $C_{glucose}$ are the initial and final glucose concentrations, respectively, and C_p is the concentration of glucose oxidation products (FoA and SoR).

At the cathode, the generated hydrogen was determined by gas chromatography (GC) using an Agilent Technologies 490 Micro gas chromatograph connected to the electrocatalytic cell. Before measurement, a stable flow rate of 40 mL min^{-1} Ar (99.999%) was bubbled for 30 mins at the cathode side of the cell. The GC product analysis was conducted every 15 min. The produced gas was identified and quantified by a calibration curve using known concentrations of standard H_2 gas. For analysis of H_2, the GC was equipped with a column containing a molecular sieve (CP-Molsieve 5 Å, Ar).[1] The FE of the gaseous product was calculated by the following equation (Eq. 4):

$$FE = \frac{n \cdot x \cdot F \cdot flow\ rate \cdot P}{R \cdot T \cdot j} \quad (4)$$

where x is the amount of gaseous product formed, P is ambient pressure (101,325 Pa), T is the ambient temperature (300 K), R is the gas constant (8.314 J K^{-1} mol^{-1}), and j is the average current density during sampling time.

4. SEM characterization

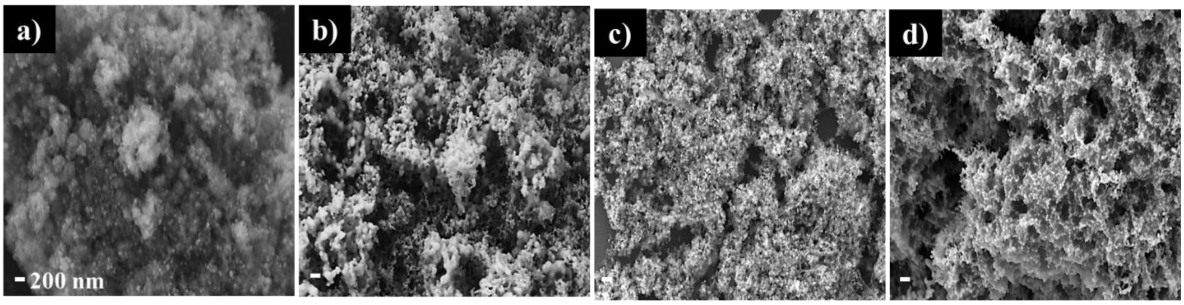

Figure S1. SEM images of a) $Cu@CoFe_2O_4$, b) $CoFeCuO_x$, c) $CoCuO_x$, and d) $FeCuO_x$ catalysts.

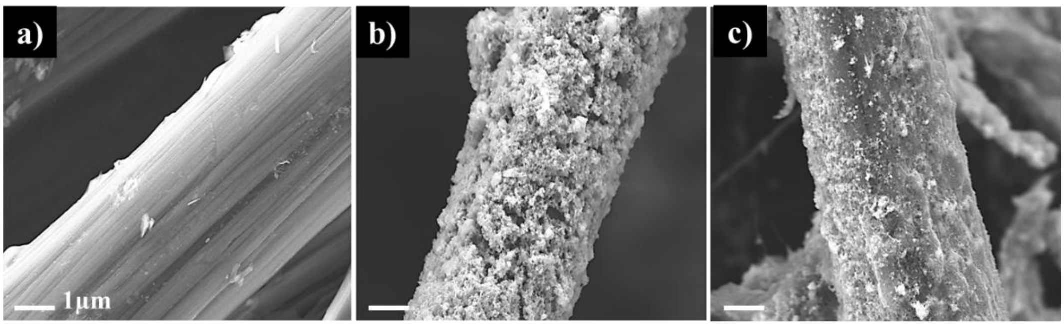

Figure S2. SEM images of electrodes a) CC, b) $Cu@CoFe_2O_4$–CC, and c) $Cu@CoFe_2O_4$–CNTs@CC**.** All scale bars correspond to 1 μm.

5. EDX and ICP characterization

Table S1. Results of the EDS and ICP-OES investigation of samples.

Sample name	SEM / EDS (atomic %)			ICP MS (atomic %)		
	Fe (%)	Co(%)	Cu(%)	Fe (%)	Co(%)	Cu(%)
CuCo		52	48		52	48
CuFe	70		30	59		41
CuFeCo	33	25	42	31	33	36
$Cu@CoFe_2O_4$	30	22	48	24	25	51

6. HRTEM Characterization

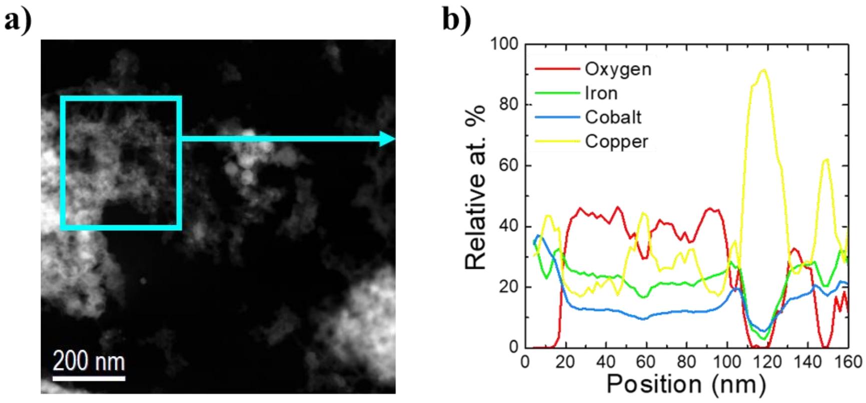

Figure S3. a) HAADF STEM image of the analyzed region of the Cu@$CoFe_2O_4$ catalyst and b) corresponding EELS line-scan profile showing the elemental distribution and Cu core enrichment.

7. XPS Characterization

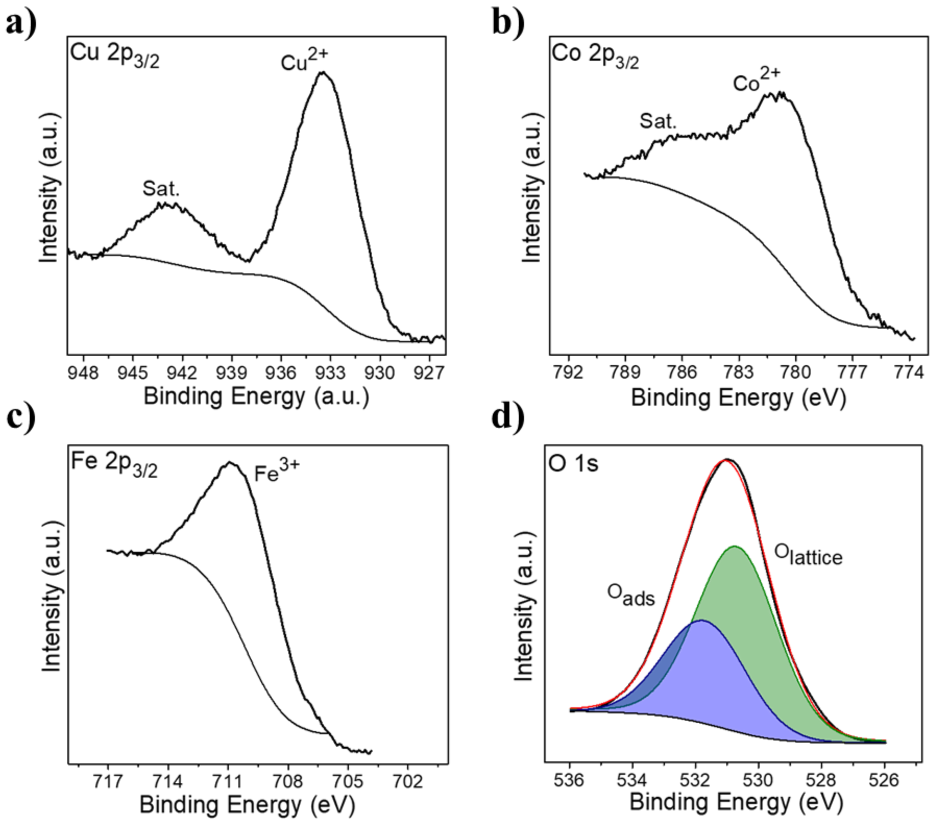

Figure S4. High-resolution XPS spectra of the Cu@$CoFe_2O_4$ catalyst. a) Cu $2p_{3/2}$, b) Co $2p_{3/2}$, c) Fe $2p_{3/2}$, and d) O 1s regions.

8. BET Characterization

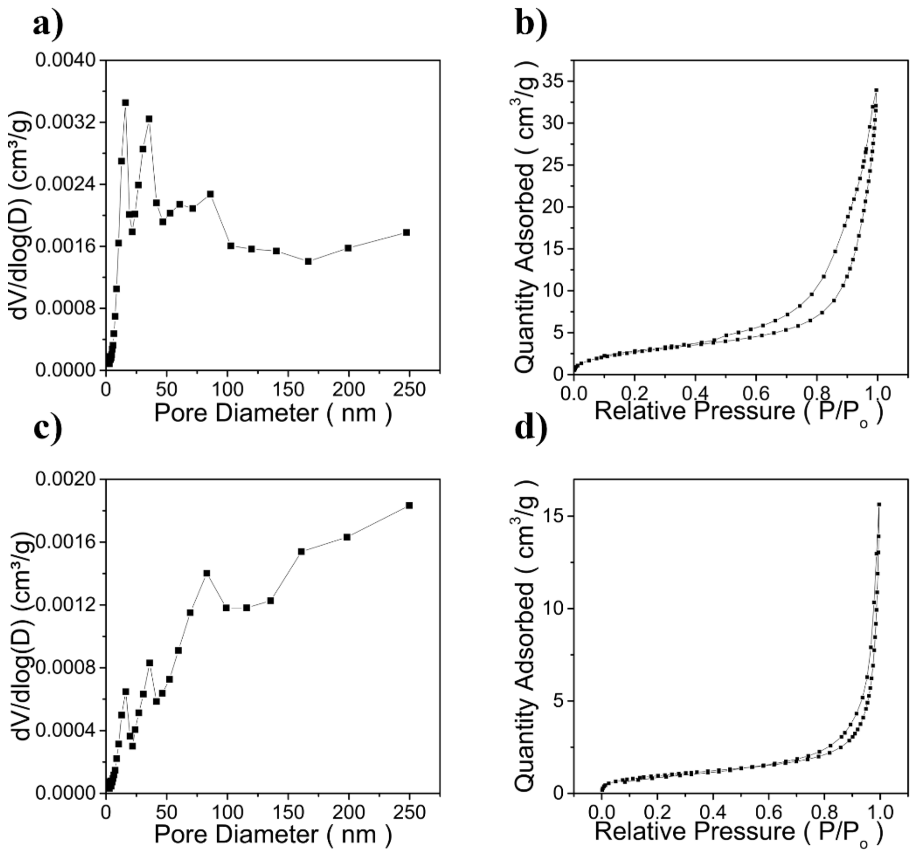

Figure S5. Nitrogen adsorption–desorption isotherms and pore size distribution of the catalytic materials, determined using the BJH method for (a–b) $CoFeCuO_x$ and (c–d) Cu@$CoFe_2O_4$.

Table S2. Textural properties of catalysts synthesized in EG obtained from nitrogen adsorption–desorption isotherms: BET surface area and BJH average pore diameter.

Alloys	Metal chloride	N_2H_4	NaOH	Mean pore size (nm)	Reference
Ni NPs	2.5 - 45 mM	0.05-0.9 M	1M	9.2	[2]
Ni NPs	0.1 M	2-12 M	1 M	2 - 600	[3]
Co–Ni–Cu	1.25 mM	0.05–0.9 M	1 M	16.4	[4]

9. Electrochemical measurements

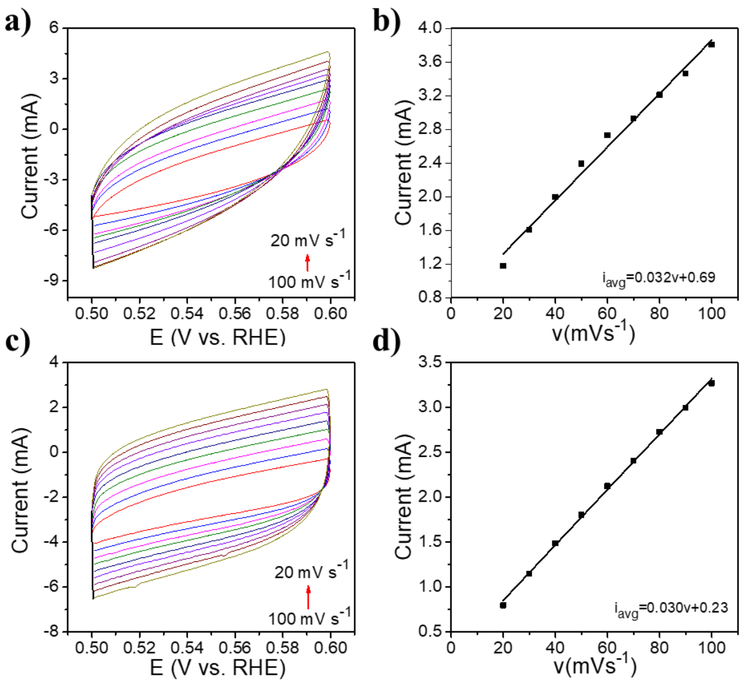

Figure S6. a-c) CVs of Cu@$CoFe_2O_4$–CNTs@CC and $CoFeCuO_x$CNTs@CC in the non-faradaic region and b-d) corresponding linear fits at 0.52 and 0.58 V.

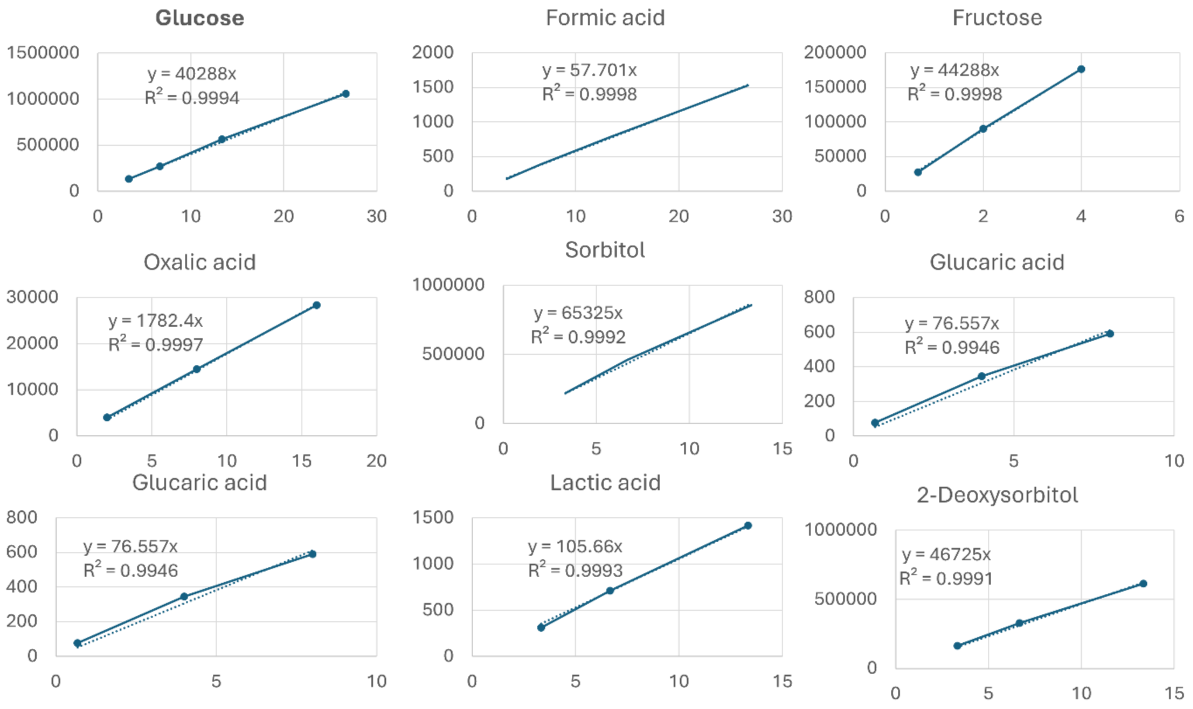

Figure S7. Calibration curves of the possible products of GOR and ECH constructed from HPLC data (x-axis: concentration, mmol L^{-1}; y-axis: HPLC peak area, a.u.).

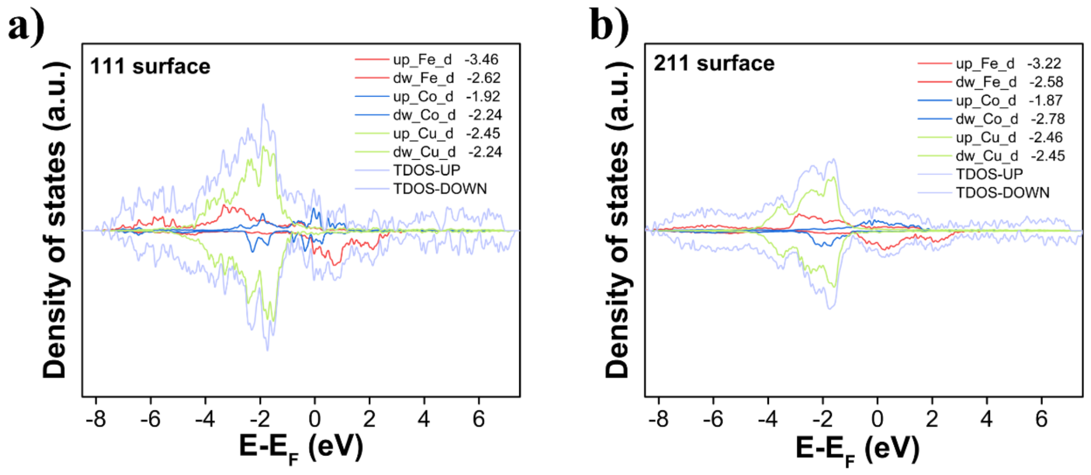

Figure S8. Spin-polarized projected density of states (PDOS) of Co, Fe, and Cu 3d orbitals for (a) (111) and (b) (211) surfaces. The dashed line indicates the Fermi level.

Table S3. Evaluation of GOR activity across various reported catalysts (C/Y/S = conversion/yield/selectivity)

Entry	Reactant	Catalyst	Main product	C/Y/S (%)	FE (%)	Potential	Electrolyte	Ref.
1	0.1 M Glucose	$NiCo_2O_4$-rGO	Gluconic acid	85.6% / -/-	74.40%	0.7 V_{RHE}	0.1 M KOH	[5]
2		NiFe-LDH	Gluconic acid	80% / -/-	60.00%	1.4 V_{RHE}		[6]
3		$NiCoSe_2$/NiOOH	Formic acid	-/90.2%/-	86.20%	1.4 V_{RHE}		[5]
4		NiFe–CoFe/NiF	Formate	-/91.6%/-	85.10%	1.4 V_{RHE}		[7]
5		CoCu oxide microspheres	Formic acid	94.4% / - / 89.7%	84.80%	1.43 V_{RHE}		[8]
8		$Ni_{75}Sn_{32}$/C	Formic acid	-/88%/-	91.00%	1.4 V_{RHE}		[9]
9		Cu_2O–Ag nanocubes	Formic acid	90% / - / 82%	85.00%	1.4 V_{RHE}		[10]
10		$CoFeNi_3/Fe_3O_4$	Formic acid	93.7% / - / 90.5%	86.20%	1.42 V_{RHE}		[11]
11		$CuCo_2O_4$ nanosheets	Formic acid	91.5% / - / 87.2%	88.90%	1.4 V_{RHE}		[8]
12		C-ZIF-EDA-$ZnNi_{0.75}$	Formic acid	-/88%/-	91%	1.40 V_{RHE}		[12]
13		Pt, Ru, Rh, Pd, Ni, Cu, and BDD (boron-doped diamond)	Sorbitol, Mannitol, Glycerol, Ethylene glycol		99% for Raney Ni	–0.5 to –0.6 V_{RHE}	0.1 M Na_2SO_4	[13]
14		Ni, Cu, Pt, Pd, Co	Sorbitol		99%	–0.5 to –1.0 V_{RHE}		[14]
**15**		**$Cu@CoFe_2O_4$–CNTs@CC**	**Sorbitol**	**>85% / – / –**	**91.2%**	**–0.4 V_{RHE}**		**This work**
			**Formic acid**	**97.5% / – / –**	**87.5%**	**1.3 V_{RHE}**	**0.1 M KOH**	

8. Reference

[1] Garcia-Villalva, R.; Biset-Peiró, M.; Alarcón, A.; Bacariza, C.; Murcia-López, S.; Guilera, J. *Comparison of methane reforming routes for hydrogen production using dielectric barrier discharge plasma-catalysis*. Int. J. Hydrogen Energy, 2024, 59, 1367–1375. https://doi.org/10.1016/j.ijhydene.2024.02.161.

[2] Wu, S. H.; Chen, D. H. *Synthesis and characterization of nickel nanoparticles by hydrazine reduction in ethylene glycol*. J. Colloid Interface Sci., 2003, 259, 282–286. https://doi.org/10.1016/S0021-9797(02)00135-2.

[3] Nik Roselina, N. R.; Azizan, A. *Ni nanoparticles: Study of particles formation and agglomeration*. Procedia Eng., 2012, 41, 1620–1626. https://doi.org/10.1016/j.proeng.2012.07.359.

[4] Singh, S.; Srivastava, P.; Singh, G. *Synthesis, characterization of Co-Ni-Cu trimetallic alloy nanocrystals and their catalytic properties, Part-91*. J. Alloys Compd., 2013, 562, 150–155. https://doi.org/10.1016/j.jallcom.2013.02.034.

[5] Lin, X.; Zhong, H.; Hu, W.; Du, J. *Nickel-Cobalt Selenide Electrocatalytic Electrode toward Glucose Oxidation Coupling with Alkaline Hydrogen Production*. Inorg. Chem., 2023, 62, 10513–10521. https://doi.org/10.1021/acs.inorgchem.3c01679.

[6] Podolean, I.; El Fergani, M.; Candu, N.; Coman, S. M.; Parvulescu, V. I. *Selective oxidation of glucose over transitional metal oxides based magnetic core-shell nanoparticles*. Catal. Today, 2023, 423, 113886. https://doi.org/10.1016/j.cattod.2022.08.028.

[7] Medrano-Banda, A. et al. *Electrochemical oxidation of glucose in alkaline environment—A comparative study of Ni and Au electrodes*. Electrochim. Acta, 2024, 487, 144159. https://doi.org/10.1016/j.electacta.2024.144159.

[8] Wu, J. et al. *Exploration of cobalt-based spinel oxide nanocatalysts MCo_2O_4 (M = Mn, Fe, Co, Ni, Cu, Zn) for glucose electrochemical sensing: $NiCo_2O_4$ exhibits largest Faradaic current*. Chem. Eng. J., 2024, 499, 156011. https://doi.org/10.1016/j.cej.2024.156011.

[9] Montaña Mora, G. et al. *Oxophilic Sn to promote glucose oxidation to formic acid in Ni nanoparticles*. ChemSusChem, 2024, 17, e202401256. https://doi.org/10.1002/cssc.202401256.

[10] Wu, X. et al. *Multi-site catalysis of high-entropy hydroxides for sustainable electrooxidation of glucose to glucaric acid*. Energy Environ. Sci., 2024, 17, 3042–3051. https://doi.org/10.1039/d4ee00221k.

[11] Shen, F.; Smith, R. L.; Li, J.; Guo, H.; Zhang, X.; Qi, X. *Critical assessment of reaction pathways for conversion of agricultural waste biomass into formic acid*. Green Chem., 2021, 23, 1536–1561 (verificar paginación). https://doi.org/10.1039/d0gc04263c.

[12] Mejia-Centeno, K. V. et al. *Glucose electrooxidation with simultaneous H_2 production on nickel-zinc electrocatalysts derived from an ethylenediamine-functionalized zeolitic imidazole framework*. Chem. Eng. J., 2025, 515, 163491. https://doi.org/10.1016/j.cej.2025.163491.

[13] Kwon, Y.; Koper, M. T. M. *Electrocatalytic hydrogenation and deoxygenation of glucose on solid metal electrodes*. ChemSusChem, 2013, 6, 455–462. https://doi.org/10.1002/cssc.201200722.

[14] Lee, J.; Jung, S.; Kim, Y. T.; Kim, H. J.; Kim, K. H. *Catalytic and electrocatalytic conversion of glucose into value-added chemicals*. Renew. Sustain. Energy Rev., 2023, 182, 113337. https://doi.org/10.1016/j.rser.2023.113337.
